# Quality of Life After Proximal Femoral Fractures Treated With Gamma Nail in Sudan

**DOI:** 10.7759/cureus.55702

**Published:** 2024-03-07

**Authors:** Hassan Mohammed Hassan Elbahri, Muhamed Ali Aydrouce Ahmed, Yousif Omer Elgaili Yousif, Hozifa Mohammed Ali Abd-Elmaged

**Affiliations:** 1 Orthopaedics and Orthopaedic Oncology, International University of Africa, Khartoum, SDN; 2 Orthopaedics and Trauma, University of Sinnar, Sinnar, SDN; 3 Surgery, Faculty of Medicine, Alzaiem Alazhari University, Khartoum, SDN; 4 Orthopaedics and Traumatology, Faculty of Medicine, Alzaiem Alazhari University, Khartoum, SDN

**Keywords:** follow-up care, orthopedic surgery, postoperative outcomes, quality of life, gamma nail, proximal femoral fractures

## Abstract

Background

Hip fracture is a public health problem globally, and it poses one of the biggest challenges in healthcare due to its associated complications.

Objectives

The aim of this study is to investigate the quality of life in adult patients in Khartoum State, Sudan, after they have undergone treatment using a gamma nail for proximal femoral fractures.

Methodology

This cross-sectional descriptive hospital-based study was conducted at Ibrahim Malik, Omdurman, and Bahri Teaching Hospitals over six months, from April to October 2022. The data were collected using an interview questionnaire that covered relevant aspects of the study. The data were analyzed using IBM SPSS Statistics for Windows, V. 26.0 (IBM Corp., Armonk, NY). The study was approved by the Sudan Medical Specialization Board, and ethical clearance was obtained.

Results

The study included 37 patients. More than half of the patients (59.5%, n=22) were women. The mean age of cases was 66.7 years (standard deviation, ±15.6). The mean time from the time of the fracture to the time of surgery was eight days (±15). Twenty-three (62.2%, n=23) (JRB1) of the patients started weight bearing on the second postoperative day. Regarding the health-related quality of life, 21.6% of the patients had a good health-related quality of life, 67.6% had a fair health-related quality of life, and 10.8% had a poor health-related quality of life. None of the patients reported an excellent quality of life. Based on the Oxford Hip Score, 54.1% of the patients had satisfactory joint function, 29.7% had mild to moderate hip joint function, 13.5% had moderate to severe hip joint function disturbance, and one patient (2.7%) had severe hip joint function problems.

Conclusion

In this study, the vast majority of the patients who underwent gamma nail surgery for hip fracture had quality of life scores in the fair to good range afterward. The results indicate that nailing is associated with good outcomes regarding quality of life and is an acceptable option for femoral fracture surgeries in Sudan.

## Introduction

Proximal femoral fracture is the most common fracture associated with fragility in the elderly, and such injuries are among the most serious healthcare problems faced by older adults [1]. As the population ages, an increased incidence of osteoporotic fragility fractures is anticipated to occur worldwide, with an annual estimate of 2.6 million proximal femoral fractures expected by 2025. Also, immobility and associated medical conditions strain health systems which are associated with proximal femoral fractures, leading to burnout [2,3]. There are various types of hip fractures, depending on the location and severity of the fracture. Intracapsular fractures and extracapsular fractures are the two main types [4], and approximately half of all proximal hip fractures are trochanteric and subtrochanteric fractures [5]. Given that these fractures may occur more frequently in the future, determining the proper treatment of these fractures is becoming increasingly crucial [6].

Hip fractures were previously fixed with rigid combinations of intramedullary nails and lateral plates, but high rates of surgical failure, metal failure, and secondary fracture displacement were reported. Subsequently, compression hip screws were used for dynamic fixation [7]. For several years, sliding hip screws have been the standard of care for hip fractures, resulting in minimal displacement and stable fractures with satisfactory outcomes. Currently, complex trochanteric fractures cannot be treated with osteosynthesis [8]. As a result, intramedullary implants are becoming increasingly popular for this type of fracture. Previously, this intramedullary device was primarily known as the GammaTM nail. Implantation of this device is minimally invasive while also providing significant biomechanical advantages [9].

In addition to reducing mortality, the management of hip fractures aims to restore patients to their prefracture level of function, minimize disability, prevent complications, and promote independence. Previous studies have evaluated various surgical techniques and implants with the primary goal of minimizing complications related to fractures and the necessity for additional surgeries. However, few studies have specifically examined the health-related quality of life of patients with proximal neck fractures [10], particularly in developing countries.

The number of Sudanese patients with hip fractures is much higher than what is reported in published papers. As life expectancy increases and osteoporosis becomes more prevalent among the elderly, the incidence of proximal femoral fractures has been on the rise. In addition, road traffic accidents among younger individuals contribute to the overall incidence of hip fractures in Sudan.

Since patient satisfaction and surgical success are closely tied to quality of life, we aimed to investigate the quality of life in patients with proximal femoral fractures who were treated with the gamma nail. Our purpose was to identify gaps in knowledge and implement appropriate interventions that would increase awareness among orthopedic surgeons, prevent mortality and morbidity, and improve the patients' quality of life. Additionally, the findings of this study will assist educational institutions in adjusting their curricula to address the identified gaps in enhancing post-surgery quality of life and preventing significant complications.

## Materials and methods

This cross-sectional descriptive study was conducted at Ibrahim Malik, Omdurman, and Bahri Teaching Hospitals in Khartoum State. These hospitals are considered to be among the most important referral centers in the state. Orthopedists at these hospitals treat patients from nearby residential and rural areas, as well as those referred from all over the country, who have experienced traumatic injuries and require orthopedic surgery.

We conducted this study between April and October 2022. All adult patients who met the inclusion criteria and had trochanteric or subtrochanteric fractures were included in the study. We selected patients aged 18 years or older with trochanteric or subtrochanteric fractures who visited the referral clinics of hospitals in the study area during the study period. Participants agreed to participate in the study and undergo six months of follow-up after surgery, and all participants had a comprehensive medical record. The study excluded patients who had multiple traumas, pathological fractures, and incomplete patient records or were bedridden due to any condition that affected their quality of life, such as paralysis or any other reason that rendered them immobile. Patients with chronic renal failure or psychological disorders were also excluded from this study.

The number of participants (n) required to form a statistically significant sample was calculated using Cochran's formula: n=(Z2pq)/d2. Z is the standard normal variable at a 5% type 1 error (p<0.05); p represents the anticipated occurrence of an event; q is equal to 1−p; and d denotes the absolute error or precision, which in this instance is 0.05% (the prevalence of trochanteric and subtrochanteric fractures as determined by the current review of management). The estimated prevalence of an event, based on consultant and hospital records, was 37. The sampling technique used was convenience sampling, which is a non-random method.

An extensive structured interview questionnaire was used to collect data for this study. The questionnaire covered all relevant aspects and variables of the study. Data were collected by the principal investigator. Clinical data and laboratory investigations were obtained from the medical records. A widely used, generic, validated, and cross-disciplinary standardized health utility instrument was employed to assess the quality of life following hip fractures. The EQ-5D-5L score and Oxford Hip Score were used.

The EQ-5D-5L is a self-rated health measure that includes a health status instrument. The analysis consists of a five-level response scale representing different levels of health: no problems, slight problems (indicating good health), moderate problems (indicating fair health), severe problems, and extreme problems (indicating poor health). The instrument also assesses five health domains related to daily activities: mobility, self-care, usual activities, pain and discomfort, and anxiety and depression.

This study encompassed a range of independent variables, including demographic characteristics, such as age, sex, education, socioeconomic status, ethnicity, and residence, as well as comorbidities. The dependent variables also included the EQ-5D-5L score, satisfaction level, and the Oxford Hip Score.

Using IBM SPSS Statistics for Windows, V. 26.0 (IBM Corp., Armonk, NY), we cleaned the data, entered it into a Microsoft Excel spreadsheet, and analyzed it. Categorical data are presented as frequencies and proportions to convey the information. Continuous data are typically presented as the mean and standard deviation. Microsoft Excel and Microsoft Word were used to create various types of graphs, including bar charts. A p-value (probability that the result is true) of <0.05 was considered statistically significant, assuming all the rules of statistical tests and the desired level of confidence. Data are represented after analysis in the form of univariate tables, cross-tabulation (bivariate tables), and figures.

The Ethical Committee of the Sudan Medical Specialization Board granted written approval and ethical clearance for this research (approval number: TA130MRE32). Written permission to conduct this study was obtained from the Ethics Committee of the Ministry of Health in Khartoum and from the administrative authorities at Ibrahim Malik, Future, Sharg Elneel, Royal Care, and Omdurman Teaching Hospitals. The data and information gathered during the study were used exclusively for research purposes. Privacy issues were intentionally addressed. A clear and simple explanation of the study was provided to all patients. Participation in the study was voluntary. Each participant had the right to withdraw at any time, and they were informed of their right to confidentiality and privacy and their right to not be harmed. The records, as well as their arrangements, were preserved. Consent was obtained from the statistical units to access the records. The precision of coronavirus disease 2019 (COVID-19) was taken into account.

## Results

A diverse group of 37 patients were included in this study. The majority (59.5%, n=22) were women, resulting in a male-to-female ratio of 2:3. The age of the patients spanned from 32 to 97 years, with the average age being 66.7 years (standard deviation of ±15.6). Notably, the age distribution had distinct peaks, with 27% of participants being between 70 and 79 years, 24.3% between 60 and 69 years, and 16.2% between 80 and 89 years. The occupational status of the patients provided valuable insights into their daily lives. An overwhelming majority of 86.5% were not currently employed, while a small percentage of 8.1% were employees, and 5.4% identified as free workers. Education levels among the participants varied substantially, with 40.5% being illiterate, 32.4% having completed primary school, 18.9% holding secondary school diplomas, and 8.1% achieving university degrees. Economic status also varied, with more than half of the patients (54.1%) falling into the middle socioeconomic bracket, 37.8% belonging to the low-income group, and 8.1% having a high socioeconomic status (Table 1).

**Table 1:** Demographics of patients undergoing orthopedic surgery

Demographics	Frequency	Percent
Age (years)		
32-39	2	5.4
40-49	4	10.8
50-59	4	10.8
60-69	9	24.3
70-79	10	27.0
80-89	6	16.2
≥90	2	5.4
Sex		
Male	15	40.5
Female	22	59.5
Occupation		
Employee	3	8.1
Free worker	2	5.4
Not working	32	86.5
Educational level		
Illiterate	15	40.5
Primary school	12	32.4
Secondary school	7	18.9
University	3	8.1
Socioeconomic status		
Low	14	37.8
Middle	20	54.1
High	3	8.1

The timing of surgeries postfracture showed interesting trends, with the mean duration being eight days (±15 days). A significant majority (75.7%) underwent surgery within seven days, while a smaller proportion (16.2%) had their surgeries between 15 and 21 days postfracture. Postoperative weight-bearing activities showed varying timelines among patients, with 13.5% starting on the first day following surgery and the majority 62.2% commencing on the second day (Table 2).

**Table 2:** Time from fracture to operation and time from operation to weight bearing in orthopedic surgery patients

	Frequency	Percent
Time from fracture to surgery (days)		
≤7	28	75.7
8-14	2	5.4
15-21	6	16.2
>30	1	2.7
Time from surgery to weight bearing		
1 day	5	13.5
2 days	23	62.2
3 days	1	2.7
3 weeks	1	2.7
1 month	4	10.8
1.5 months	1	2.7
2 months	1	2.7
3 months	1	2.7

Trauma types leading to fractures were predominantly falls (83.8%), while road traffic accidents accounted for a smaller yet still notable portion (16.2%). Post-surgical complications occurred in an overwhelming majority of patients (91.9%), with infection and delayed union being reported in individual cases. Patient satisfaction levels revealed that only 27% were even partially satisfied, while the majority (73%) expressed dissatisfaction with their treatment outcomes.

Health-related quality of life assessments demonstrated a spectrum of outcomes, with 21.6% reporting good quality of life, 67.6% experiencing fair quality of life, and 10.8% facing challenges with poor quality of life. Evaluation based on the Oxford Hip Score highlighted various levels of joint function satisfaction among patients, with 54.1% reporting satisfactory joint function, 29.7% experiencing mild to moderate issues, 13.5% dealing with moderate to severe complications, and a small fraction (2.7%) facing severe hip joint function challenges (Table 3).

**Table 3:** Health-related quality of life and Oxford Hip Score in orthopedic surgery patients

	Frequency	Percent
Health-related quality of life		
Good	8	21.6
Fair	25	67.6
Poor	4	10.8
Oxford Hip Score		
0 to <20, severe hip arthritis	1	2.7
20 to <30, moderate to severe hip arthritis	5	13.5
30 to <40, mild to moderate hip arthritis	11	29.7
≥40, satisfactory joint function	20	54.1

## Discussion

Multiple studies have investigated the quality of life among patients after femoral fracture surgeries. However, very few studies have specifically focused on gamma nail surgeries. Moreover, no similar work has been conducted in Sudan on this issue, making the current study the first of its kind.

The majority of participants in this study were women, which is consistent with a previous study conducted in Germany, Austria, and Switzerland [11]. However, in a previous study conducted in Turkey, the majority of participants were male [12]. This difference could be explained by the variation in the age groups of the study participants. Similar to the European study and its focus on geriatric individuals, the majority of our participants were elderly women who were likely experiencing postmenopausal osteoporotic changes.

The majority of patients in our study did not work after surgery; however, this could be explained by the older age of the participants. The advanced age of our study population also accounts for the low percentage of road traffic accidents as a mode of trauma in this study. It is well known that road traffic accidents and high-energy fractures are the primary causes of hip fractures in young adults [13].

The vast majority of patients in this study had fair to good quality of life scores, but none reported an excellent quality of life. In a previous European study, participants who underwent cephalomedullary nail surgeries had a mean score in the good to excellent range, with none reporting a poor quality of life [11]. In that study, participants who underwent cephalomedullary nail surgeries had a significantly higher quality of life than those who underwent sliding hip screw surgeries. Moreover, a study conducted in Malawi showed that patients treated with intramedullary nails experienced a good to excellent quality of life, which continued to improve in the months following surgery [14]. In that study, a comparison was made between skeletal traction and intramedullary nailing, and no difference in the quality of life was reported between the two procedures. These results indicate that internal fixation with nails is associated with positive outcomes and is a viable option for femoral fracture surgeries.

Based on the Oxford Hip Score, the majority of patients in our study had satisfactory joint function or mild to moderate arthritis. However, based on the patients' self-reported satisfaction with their quality of life after surgery, almost three-quarters of them were unsatisfied. Similarly, in a previous study conducted in Austria, excellent to good joint function was reported after gamma nail treatment [15]. Moreover, a European study reported that patients treated with cephalomedullary nails had a better ability to walk 120 days after surgery compared with those treated with sliding hip screws [11]. However, a Turkish study reported comparable mobility scores among elderly patients treated with proximal femoral nail and cementless bipolar hemiarthroplasty [12]. Moreover, in this study, the majority of patients began weight bearing within the first two days after surgery, and the overwhelming majority of them encountered no complications. These findings suggest that treating hip fractures with nails leads to positive outcomes in hip function.

This study was limited by the small sample size and the inclusion of patients from only one hospital. This limitation was due to the lack of facilities and personnel required to collect data from a larger number of patients and multiple centers. These factors make it challenging to generalize our findings. Moreover, the lack of prior research in Sudan makes it difficult to compare the acquired data and draw conclusions. Another limitation is the absence of baseline information on patients' quality of life, which could be used for comparison with post-surgery data to determine the impact of surgery on patients' quality of life.

Based on our results, we recommend conducting additional research with larger sample sizes and involving patients from multiple hospitals to collect more precise data on the outcomes of hip fractures treated with gamma nails. This future research should focus on assessing the impact on quality of life and hip joint function. Furthermore, studies should use similar or comparable designs to facilitate data comparison and draw meaningful conclusions. Additionally, studies should include groups treated with different procedures to compare the outcomes of various fixation techniques. This approach will help raise doctors' awareness about the effects of different procedures on patients' quality of life and joint function, as well as highlight any differences between them.

## Conclusions

The vast majority of patients who underwent gamma nail surgery for hip fracture had fair to good quality of life scores, although none reported an excellent quality of life. Nailing is associated with positive outcomes in terms of quality of life and is a viable option for femoral fracture surgeries.

The majority of patients had satisfactory joint function or mild to moderate arthritis. Additionally, most of them were able to start weight bearing within the first two days of surgery, and the vast majority experienced no complications. These findings suggest that treating hip fractures with nails leads to positive outcomes in hip function.

The majority of participants with hip fractures were elderly women who experienced a fall as a mode of trauma. Their fractures could be attributed to postmenopausal osteoporotic changes.
